# The Nuclear Export Receptors TbMex67 and TbMtr2 Are Required for Ribosome Biogenesis in Trypanosoma brucei

**DOI:** 10.1128/mSphere.00343-19

**Published:** 2019-07-03

**Authors:** Constance Rink, Martin Ciganda, Noreen Williams

**Affiliations:** a Department of Microbiology and Immunology, University at Buffalo, Buffalo, New York, USA; Carnegie Mellon University

**Keywords:** nuclear export, protein-RNA interactions, protein-protein interactions, ribosome biogenesis

## Abstract

Ribosomal maturation is a complex and highly conserved biological process involving migration of a continuously changing RNP across multiple cellular compartments. A critical point in this process is the translocation of individual ribosomal subunits (60S and 40S) from the nucleus to the cytoplasm, and a number of export factors participate in this process. In this study, we characterize the functional role of the auxiliary export receptors TbMex67 and TbMtr2 in ribosome biogenesis in the parasite Trypanosoma brucei. We demonstrate that depletion of each of these proteins dramatically impacts the steady-state levels of other proteins involved in ribosome biogenesis, including the trypanosome-specific factors P34 and P37. In addition, we observe that the loss of TbMex67 or TbMtr2 leads to aberrant ribosome formation, rRNA processing, and polysome formation. Although the TbMex67-TbMtr2 heterodimer is structurally distinct from Mex67-Mtr2 complexes previously studied, our data show that they retain a conserved function in ribosome biogenesis.

**IMPORTANCE** The nuclear export of ribosomal subunits (60S and 40S) depends in part on the activity of the essential auxiliary export receptors TbMtr2 and TbMex67. When these proteins are individually depleted from the medically and agriculturally significant parasite Trypanosoma brucei, distinct alterations in the processing of the rRNAs of the large subunit (60S) are observed as well as aberrations in the assembly of functional ribosomes (polysomes). We also established that TbMex67 and TbMtr2 interact directly or indirectly with the protein components of the 5S RNP, including the trypanosome-specific P34/P37. The critical role that TbMex67 and TbMtr2 play in this essential biological process together with their parasite-specific interactions may provide new therapeutic targets against this important parasite.

## Abstract (precis)

The nuclear export of ribosomal subunits (60S and 40S) depends in part on the activity of the essential auxiliary export receptors TbMtr2 and TbMex67. When these proteins are individually depleted from the medically and agriculturally significant parasite Trypanosoma brucei, distinct alterations in the processing of the rRNAs of the large subunit (60S) are observed as well as aberrations in the assembly of functional ribosomes (polysomes). We also established that TbMex67 and TbMtr2 interact directly or indirectly with the protein components of the 5S RNP, including the trypanosome-specific P34/P37. The critical role that TbMex67 and TbMtr2 play in this essential biological process together with their parasite-specific interactions may provide new therapeutic targets against this important parasite.

## INTRODUCTION

African trypanosomiasis affects both humans and animals and is caused by the protozoan parasite Trypanosoma brucei. The disease has significantly impacted sub-Saharan Africa for millennia. Approximately 60 to 70 million people are at risk of contracting human African trypanosomiasis (HAT; also called African sleeping sickness), while over 60 million livestock are at risk of contracting animal trypanosomiasis (AAT; also called nagana) (1, 2). HAT is fatal if left untreated, although drug treatments, vector control, and other preventative measures have led to a decrease in mortality in recent years. Nagana (the animal trypanosomiasis) results in significant economic impact to the region with an estimated annual loss of $4.5 billion (2). The current treatments are inadequate due to toxicity, cost, and drug resistance (3). Targeted development of new, more effective chemotherapeutics requires a better understanding of essential parasite-specific biological processes.

Ribosome biogenesis is a highly conserved and vital process for all living cells, including T. brucei. Ribosome assembly in Saccharomyces cerevisiae involves more than 80 ribosomal proteins, over 200 nonribosomal proteins, and 4 species of rRNA (28S, 18S, 5.8S, and 5S) (4). A critical step in ribosome biogenesis is the transport of the pre-60S and pre-40S particles across the nuclear pore by the nuclear export complex (NEC), which includes the exportin 1 (Xpo1) and Nmd3 proteins (4). Additional auxiliary export receptors Mex67 and Mtr2 facilitate the transport of ribosomal subunits through the nuclear pore complex (NPC) (4, 5). As a heterodimer (Mex67-Mtr2), these proteins bind to the phenylalanine-glycine-rich repeat regions (FG) in the nucleoporins lining the nucleopore, thus allowing easy passage of the ribosomal subunits (4, 5).

Mex67 and Mtr2 are non-importin-beta nuclear export receptors that are involved in the transport of mRNA, ribosomal subunits, and tRNA across the NPC (4 – 6). The basic structure of Mex67 is comprised of an N-terminal leucine-rich repeat (LRR), followed by a nuclear transport factor 2-like (NTF2-like) domain and a C-terminal ubiquitin-associated (UBA) domain (7). Mtr2 contains only an NTF2-like domain (8). Mex67 and Mtr2 form a heterodimer through direct interaction of their NTF2-like domains (7, 9).

The mechanism by which the Mex67-Mtr2 complex associates with its cargo has been examined predominantly in S. cerevisiae. Mex67-Mtr2 association with the pre-60S subunit was originally hypothesized to be through the direct interaction of 5S rRNA with positively charged amino acids within an extended loop region of the NTF2-like domain of Mex67 (10). Association with the 40S ribosomal subunit occurs via this same extended loop region in ScMex67 together with the C-terminal UBA domain (11). More recent *in vivo* work in yeast has suggested that ScMex67-ScMtr2 binds to solvent-exposed regions of both the 28S rRNA and 5.8S rRNA of the 60S ribosomal subunit (12).

In T. brucei, orthologues of Mex67 (TbMex67) and Mtr2 (TbMtr2) have been identified and shown to participate in mRNA export (13, 14). Similarly to other eukaryotic orthologues, TbMex67 has been shown to associate with several nuclear pore proteins (15). However, TbMex67 is structurally distinct compared to other orthologues. At its N terminus, TbMex67 contains a zinc finger motif rather than the RNA recognition motif (RRM) present in other orthologues, followed by a leucine-rich repeat (LRR) domain and NTF2-like domain. At the C terminus, TbMex67 lacks a canonical UBA domain present in other orthologues. The N-terminal zinc finger motif of TbMex67 was shown to be essential in mRNA export in trypanosomes (13, 14). TbMtr2 is similar to other orthologues containing only an NTF2-like domain. The structural differences found within TbMex67 raise the question of whether TbMex67 and TbMtr2 may or may not share the many functions found for these proteins in other organisms. In this study, we examine the role of TbMex67 and TbMtr2 and demonstrate that they also function in the production of functional ribosomes.

## RESULTS

### TbMtr2 and TbMex67 are essential proteins in T. brucei.

**FIG 1:**
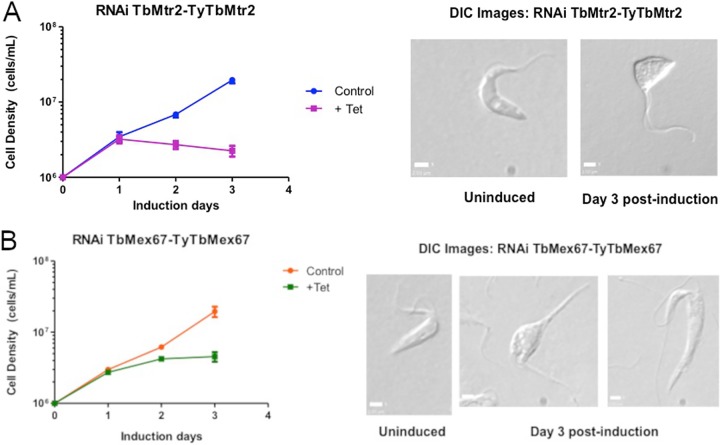
TbMtr2 and TbMex67 are essential proteins in T. brucei. RNAi TbMtr2-TyTbMtr2 (A) and RNAi TbMex67-TyTbMex67 (B) cells were treated with tetracycline (+ Tet) to induce the degradation of TbMtr2 or TbMex67 transcript within the cells over the course of 3 days. Cell density of induced versus uninduced RNAi cells was plotted over time based on an average from three biological replicates. Differential interference contrast (DIC) images were taken of TbMtr2 and TbMex67 RNAi cells when uninduced and at day 3 postinduction.

Previous studies in T. brucei have demonstrated that the depletion of the nuclear export proteins TbMtr2 and TbMex67 results in significant growth defects and is necessary for the export of mRNA (13, 14). However, that work did not address the impact of TbMex67 and TbMtr2 on rRNA metabolism and function. To address this question, we created a T. brucei 29-13 procyclic cell line containing an endogenous Ty-tagged TbMtr2 expressed in an RNA interference (RNAi)-TbMtr2 background targeting the full-length protein and a second cell line containing an endogenous Ty-tagged TbMex67 expressed in an RNAi-TbMex67 background targeting the 3′ untranslated region (UTR) of TbMex67. In accordance with data previously obtained by Dostalova et al. (13), our results show that upon induction of RNAi targeting either TbMtr2 or TbMex67, a significant growth defect is observed (Fig. 1A and B). Microscopic analysis of the two cell lines showed that a majority of the cells experienced dramatic changes in morphology with cell rounding (Fig. 1A and B) upon induction of RNAi. Depletion of TbMtr2 led to a more rapid impact on growth and morphology than the loss of TbMex67. In the case of the TbMtr2 RNAi parasites, a sharp drop in cell growth was observed after day 1 of induction while TbMex67 RNAi parasites showed only a slight decrease in cell growth. Changes in cell morphology were observed as early as day 1 postinduction in TbMtr2 RNAi parasites, while in TbMex67 RNAi parasites the cell rounding was apparent at day 2 postinduction. In both cases, slight elongation of the cells was followed by rounding and, in some cases, impairment in cell division. However, the loss of either protein resulted in significantly altered morphology and inhibition of growth. For completion of growth analysis, parasites were grown in culture until 5 days postinduction (data not shown), and cessation of growth was noted by the lack of cell movement, along with the significant abnormal cell morphology stated above.

### Association of TbMex67 and TbMtr2 *in vivo* with the components of the 5S RNP.

**FIG 2:**
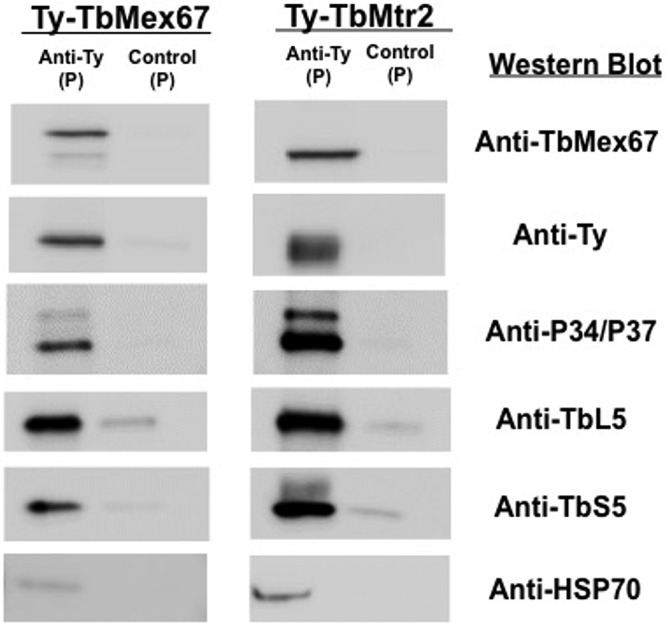
TbMex67 associates with TbMtr2 and is found within a complex containing 5S RNPs *in vivo*. Immune capture was performed (anti-Ty) with whole-cell extracts of RNAi TbMtr2-TyTbMtr2 (uninduced) and Ty-TbMex67 procyclic cell lines. For each immune capture, pellet (P) and bead-alone control (P) are shown. Results shown are representative of three biological replicates.

Previous work in S. cerevisiae had shown that ScMex67 and ScMtr2 interacted with the 60S ribosomal subunit (10). Early reports suggested that the interaction was through the 5S rRNA, while a later report suggested that the interaction was through 25/28S and/or 5.8S rRNA (10, 12). Data from other experiments in our laboratory using 5S rRNA affinity capture supported a possible interaction with TbMex67 (data not shown), although those results were not definitive. To determine whether TbMex67 and TbMtr2 interact with the 60S ribosomal subunit and whether that might occur through 5S rRNA, we performed initial experiments with trypanosome procyclic cell lines expressing Ty-tagged TbMex67 (Ty-TbMex67) or Ty-tagged TbMtr2 (RNAi TbMtr2-TyTbMtr2, uninduced cells). We performed affinity capture for TbMex67 and TbMtr2 via the Ty tag followed by Western blot analyses to determine interacting proteins. Results showed that the 5S RNP components TbL5 and P34/P37 associated with the tagged TbMex67 and TbMtr2 proteins (Fig. 2). We observed very little nonspecific interaction with the anti-Ty beads as shown by the Hsp70 negative control for both Ty-TbMex67 and Ty-TbMtr2.

### The depletion of TbMtr2 leads to a decrease in TbMex67 and 5S RNPs.

**FIG 3:**
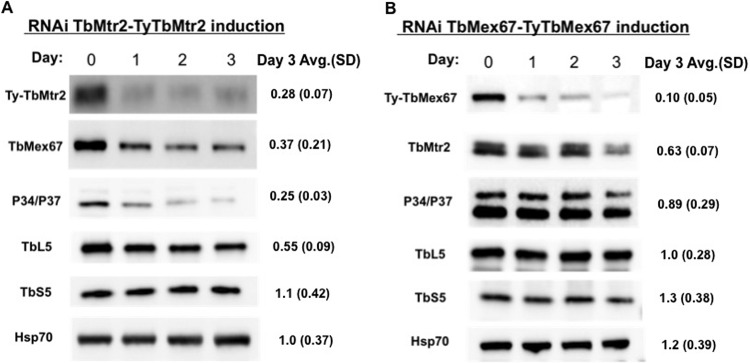
The depletion of TbMtr2 or TbMex67 alters steady-state levels of TbMex67 and specific 5S RNPs. TbMtr2 (A) or TbMex67 (B) RNAi cells were collected daily and analyzed by SDS-PAGE. Western blot analysis was performed using either epitope tag or protein-specific antibodies to examine protein levels. Hsp70 protein was used as a loading control for these experiments. Quantification of day 3 levels relative to day 0 with standard deviations was based on at least three biological replicates.

With the observation of a significant growth defect with the loss of TbMtr2 protein and an association of the 5S RNPs, we were interested in determining the impact of TbMtr2 on its binding partner TbMex67 and other ribosomal and ribosome-associated proteins. We collected cells daily following RNAi induction and performed Western blot analysis with antibodies specific for pre-60S subunit-associated proteins (TbMex67, TbL5, and P34/P37) and a 40S subunit protein (TbS5). As expected, we observed TbMtr2 steady-state protein levels drop to 0.28 (standard deviation [SD], 0.07) of uninduced levels by the 3rd day of RNAi induction (uninduced [day 0] level is set as 1.0). We found that its binding partner, TbMex67, also is substantially decreased in steady-state protein levels when TbMtr2 is knocked down (0.37 [SD, 0.21]) (Fig. 3A), consistent with the hypothesis that the formation of the heterodimer is required to stabilize TbMex67. The loss of TbMtr2 also impacts the steady-state levels of two other proteins analyzed (P34/P37 and TbL5) to different extents. We found that the trypanosome-specific factors P34 and P37 experienced the greatest impact upon depletion of TbMtr2 with only 0.25 (SD, 0.03) remaining by day 3 postinduction. The levels of conserved ribosomal protein TbL5 decreased, although not as dramatically (to 0.55 [SD, 0.09]). The 40S subunit protein TbS5 did not exhibit a decrease (1.1 [SD, 0.42]), suggesting that loss of the TbMtr2-TbMex67 heterodimer specifically impacted the 60S components.

### The depletion of TbMex67 has a moderate impact on 5S RNPs.

In the case of the TbMex67 RNAi cell line, we saw a 90% decrease in TbMex67 by day 3 postinduction (Fig. 3B). Similarly to our results observed in the TbMtr2 RNAi cell line, the corresponding binding partner was also decreased. In TbMex67-depleted parasites, TbMtr2 was decreased to 0.63 (SD, 0.07). However, when we examined the steady-state levels of the other interacting proteins examined, we saw significantly less impact than we had for the loss of TbMtr2. Only P34/P37, which was decreased to 0.89 (SD, 0.29) of the uninduced levels by day 3 postinduction, was significantly reduced. TbL5 (1.0 [SD, 0.28]) and TbS5 (1.3 [SD, 0.38]) showed no reduction.

### Loss of TbMtr2 and TbMex67 does not lead to a significant change in steady-state mature rRNAs.

**FIG 4:**
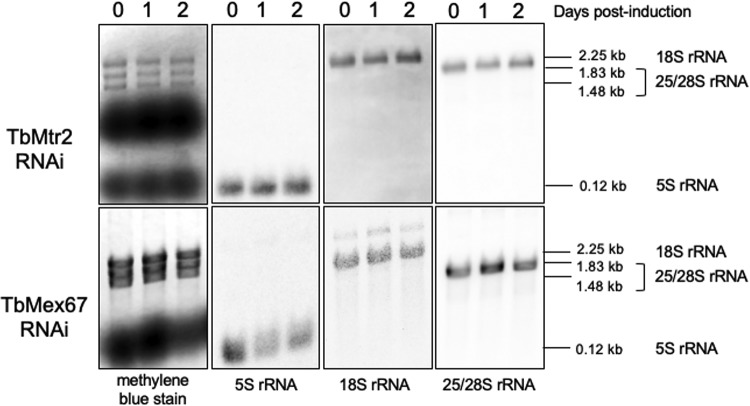
Depletion of TbMtr2 and TbMex67 does not disrupt steady-state levels of mature rRNAs. Total RNA was extracted from cells throughout an induction course and analyzed by Northern blotting with probes designed to identify fully processed rRNAs. Molecular sizes are indicated on the right. At least three biological replicates were analyzed, and representative results are shown.

We examined the mature rRNAs by Northern blotting with probes directed against completely processed 18S, 25/28S, and 5S and found that their steady-state levels were not altered upon induction for the TbMtr2 RNAi cell line (Fig. 4, top panel). In the case of TbMex67, the steady-state levels of 5S rRNA were altered upon depletion of the protein. In this case, we observed a 2-fold decrease of the 5S rRNA levels compared to the steady-state levels of 25/28S rRNA by day 2 (Fig. 4, bottom panel).

### Loss of TbMtr2 and TbMex67 leads to an overall decrease in translation.

**FIG 5:**
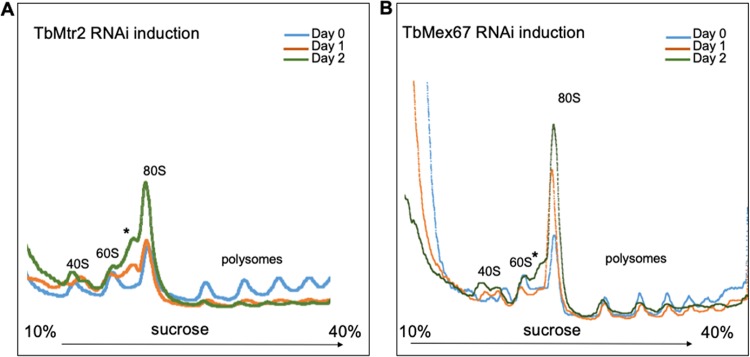
Depletion of TbMtr2 and TbMex67 disrupts ribosomal biogenesis. Cycloheximide-treated extracts from cells collected on days 0, 1, and 2 postinduction were fractionated by ultracentrifugation on 10% to 40% sucrose gradients. The figure indicates the position of the ribosomal subunits, the 80S ribosome, and the polysomes. An asterisk marks the position of a peak seen upon loss of TbMtr2 (A) or TbMex67 (B). At least three biological replicates were analyzed, and representative results are shown.

To further investigate the phenotypic effects of the specific depletion of TbMtr2 and TbMex67 on the process of ribosome biogenesis, we first analyzed cycloheximide-treated extracts from cells obtained at increasing time points after the induction of RNAi targeting TbMtr2. Upon loss of TbMtr2, we observed an overall decrease in the relative abundance of polysomes compared to 80S monosomes. As shown in Fig. 5, the area under the peaks that corresponds to polysomes, i.e., particles of increasing numbers of ribosomes actively engaged in the translation of a given mRNA, decreases upon induction of RNAi targeting either TbMtr2 (Fig. 5A) or TbMex67 (Fig. 5B) when quantified relative to the 80S peak and normalized to day 0. This effect was evident as early as the 1st day postinduction and became more pronounced as the induction progressed. The fact that we were able to observe this shift in the profile early in the induction indicates that this is a specific effect, rather than an indirect effect resulting from deleterious processes elsewhere in the cell. After 24 h of TbMtr2 RNAi induction, the polysome/80S ratio was decreased to 0.48 (SD, 0.11) relative to uninduced cells, and by day 2 this ratio was further reduced to 0.28 (SD, 0.12). In the case of TbMex67, the polysome/80S ratio dropped to 0.89 (SD, 0.24) of uninduced cells after 24 h of tetracycline treatment and to a further 0.53 (SD, 0.07) on day 2 of induction.

### Loss of TbMtr2 and TbMex67 leads to alterations in the composition of ribosomal subunits.

In addition to the effect on the abundance of polysomes, our experiments revealed that loss of either TbMtr2 or TbMex67 leads to changes in the composition of ribosomal subunits. As shown in Fig. 5, uninduced cells exhibit a typical distribution of 40S and 60S peaks in the less-dense section of the gradient, similarly to wild-type procyclic cells (16). Northern blot analysis of these 40S and 60S peaks confirmed that they contained the expected 18S and 25/28S rRNAs, respectively (data not shown). However, as early as day 1 after induction of the RNAi, the depletion of TbMtr2 or TbMex67 results in the appearance of an additional peak of intermediate density between the 60S peak and the 80S particle (Fig. 5A and B, asterisk). The population of particles of this size increased as time of induction progressed, as shown by the increased area under the peak on days 2 and 3.

### Depletion of TbMtr2 and TbMex67 interferes with normal processing of rRNAs.

**FIG 6:**
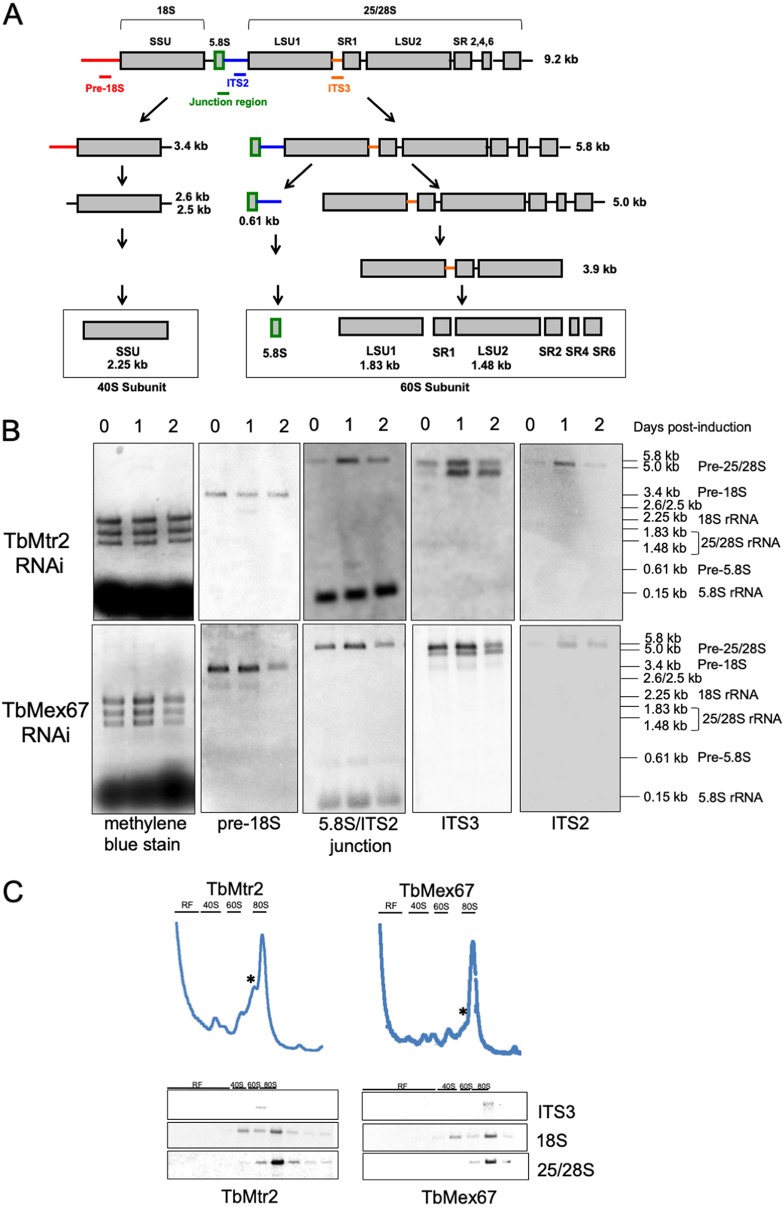
(A) The rRNA processing pathway in trypanosomes and the probes used to detect intermediates in the pathway. (Republished from reference 20.) (B) Depletion of TbMtr2 and TbMex67 interferes with normal processing of rRNAs. Total RNA was extracted from cells throughout an induction course and analyzed by Northern blotting with probes designed to identify intermediates. Molecular sizes are indicated on the right. At least three biological replicates were analyzed, and representative results are shown. (C) A 25/28S processing intermediate is a constituent of aberrant particles elicited by the depletion of TbMtr2 and TbMex67. RNA was purified from fractions from polysomal gradients obtained from day 2 postinduction extracts and analyzed by Northern blotting. The peak marked with an asterisk (cf. Fig. 5) contains an RNA that reacts with the ITS3 probe. At least three biological replicates were analyzed, and representative results are shown.

The results from the polysomal profiles prompted us to further investigate the effects that loss of TbMtr2 or TbMex67 has on the rRNA processing pathway. We employed a set of probes designed to specifically hybridize with both mature rRNAs and immature processing intermediates to detect these species by Northern blotting as the induction of TbMtr2 progressed. As illustrated in Fig. 6A, normal processing of the rRNA precursor (9.2-kb species) involves an early cleavage event that branches the processing pathway into two main arms, one of which leads to the formation of mature 18S rRNA. The intermediates of this branch of the pathway are 3.4 kb and 2.6 kb long, and they can be identified using a probe that hybridizes at the 5′ terminus of this pre-18S intermediate (pre-18S probe). The second branch of the pathway starts with a 5.8-kb intermediate (which reacts with probes directed against the 5.8S/ITS2 junction, ITS2, and ITS3). An internal cleavage event at the ITS2 site of this 5.8-kb intermediate branches the pathway a second time, generating an 0.61-kb intermediate and a 5.0-kb intermediate. The 0.61-kb intermediate is a precursor of 5.8S rRNA and can be detected with the 5.8S/ITS2 junction probe. The 5.0-kb intermediate is a precursor of 25/28S rRNA and reacts with the ITS3 probes.

We first examined the pathway that leads to the formation of 18S rRNA using the pre-18S probe (Fig. 6B). No accumulation of the 3.4-kb precursor is detected following induction. However, the ∼2.6-kb product, resulting from further processing of the 3.4-kb intermediate, can be faintly but consistently detected upon induction of the RNAi for TbMtr2. By the 2nd day of induction, this product is no longer detectable, suggesting that the cells are partially recovering from this specific event.

Analysis of the processing intermediates of the 5.8S pathway showed that induction of the TbMtr2 or TbMex67 RNAi leads to an accumulation of the 5.8-kb species and an increase (30% more on day 1 than on day 0) in the abundance of the 0.61-kb species. We also detected a more subtle but consistent effect on the size distribution of the 5.8S mature rRNA (Fig. 6B). The mature size of 5.8S rRNA is achieved by exonucleolytic processing of the 0.61-kb intermediate to yield an ∼0.5-kb mature product. Upon induction of TbMtr2 RNAi, we observe final maturation products of higher molecular weight, indicating a processing defect. The effect is less pronounced for our TbMex67 RNAi cell line.

Finally, we turned to the branch of the processing pathway that leads to the formation of mature 25/28S rRNA. Processing of the 5.8-kb precursor by cleavage in the intervening sequence between the 5.8S rRNA sequence and the 25/28S rRNA sequence leads to the formation of the 5.0-kb precursor. This precursor, detected with probe ITS3, was also found to accumulate upon loss of TbMtr2 or TbMex67 (Fig. 6B). The 5.8-kb intermediate is the same species detected in the fraction of the sucrose gradient corresponding to the peak that appears upon loss of TbMtr2 and TbMex67 (Fig. 6C, marked with an asterisk). The probe directed against ITS3 can detect both the 5.8-kb and 5.0-kb intermediates. Quantification of the relative abundance of these two species allowed us to determine that the ratio of the 5.8-kb signal to the 5.0-kb signal is 1.8 in uninduced TbMtr2 and TbMex67 cells. This may indicate that the cleavage in the intervening sequence that separates the 5.8S precursor from the 25/28S precursor occurs with lower kinetics and that further processing of the 5.0-kb product (25.5S in yeast) is more rapid. However, loss of TbMtr2 or TbMex67 leads to a reversal in the ratio of these products. By day 3 postinduction, the 5.8-kb to 5.0-kb ratio is 0.7, suggesting that the rapid processing of the 5.0-kb intermediate has stalled under these conditions. Overall, our data indicate that depletion of either TbMtr2 or TbMex67 prevents normal processing of rRNAs, especially those of the 60S subunit, and that accumulation of these intermediates alters the normal composition of ribosomal subunit particles and likely contributes to an overall decrease in translational activity.

## DISCUSSION

An essential and central step in the life cycle of T. brucei involves a metabolic switch between quiescent, nonreplicating cells (metacyclic salivary forms in the tsetse fly or bloodstream stumpy cells in circulation) and actively replicating cells (bloodstream slender forms in circulation or procyclic cells in the gut of the insect) (17, 18). This transition requires an increase in protein synthesis. Traditionally, the control for this process is translation initiation (19). However, nuclear export of translationally competent 60S and 40S preribosomal particles is also essential for this process. Studies from the model system S. cerevisiae have shown that the transport of several distinct classes of RNPs containing mRNAs, tRNAs, and rRNAs to the eukaryotic cytoplasm through the nuclear pore involves the Mex67-Mtr2 heterodimer (6, 10, 11). The presence of unique structural elements in T. brucei Mex67 (such as a zinc finger motif) which are essential for mRNA export strongly argues for unique function(s). Our work demonstrates that TbMex67 and TbMtr2 participate in the process of ribosome biogenesis, a process in which trypanosome-specific factors have been described to participate, associating with preribosomal particles as part of their maturation and transport to the cytoplasm (20).

In T. brucei, ribosome nuclear export has not been well characterized. Previous work has identified and characterized the homologues of the conserved export proteins TbXpo1 and TbNmd3 (21 – 23). We showed that the trypanosome-specific proteins P34 and P37, which form a preribosomal complex with the conserved ribosomal protein L5 and 5S rRNA in the nucleoplasm (24, 25), also associate with both early and late maturation stages of the large ribosomal subunit (60S) (21). Significantly, reduced expression of P34 and P37 resulted in a loss of association between the pre-60S particle and the nuclear export complex TbXpo1-TbNmd3 (21). Our data suggested a potential direct interaction between P34/P37 and the nuclear export complex. This led us to ask whether TbMex67 and TbMtr2 might also be involved in 60S subunit export and interact with the trypanosome-specific proteins P34 and P37.

Our approach to examining the function of the TbMex67-TbMtr2 complex was to deplete the cells of each protein individually and determine the impact on ribosome biogenesis. Using RNA interference directed against TbMex67 and TbMtr2 individually, we found that both proteins are essential for the growth of T. brucei as had previously been shown (Fig. 1A and B) (13, 14). We also observed significant morphological changes in the parasites lacking TbMex67 or TbMtr2 as early as the 1st day postinduction (Fig. 1A and B). We next examined the interactions of the TbMex67-TbMtr2 complex using an affinity capture approach. Ty-tagged TbMex67 or Ty-tagged TbMtr2 was expressed in trypanosomes and affinity captured. Results from both cell lines (Fig. 2) showed association of the complementary member of the complex (TbMex67 or TbMtr2) as well as the protein components of the 5S RNP (L5 and P34/P37). We then looked at the effect of loss of each protein on the steady-state levels of the other complex partners. In both cases, the loss of one member of the TbMex67-TbMtr2 complex led to a loss in the other binding partner (Fig. 3). The effect on steady-state protein levels of the partner protein that we observed was more pronounced in parasites depleted of TbMtr2 than parasites depleted of TbMex67, even though the levels of TbMex67 were more strongly decreased in its RNAi cells. This result may be because the formation of the heterodimer is required to stabilize Mex67, as has been found in yeast (7). The differential stabilities of TbMtr2 and TbMex67 outside the heterodimer may also suggest different functions beyond the heterodimer.

We also analyzed the levels of other proteins that might interact with the TbMex67-TbMtr2 complex in ribosomal subunit export. Loss of TbMtr2 led to a significant decrease in P34/P37 (to 0.25 of uninduced levels) and had a significant impact on the 60S ribosomal protein TbL5 but no impact on 40S ribosomal protein S5 (Fig. 3A). However, when TbMex67 is depleted from T. brucei, we observe that the relative protein levels of trypanosome-specific P34/P37 are modestly decreased (0.89) (Fig. 3B). Further, the other proteins, TbL5 and TbS5, were unchanged. This may suggest that TbMtr2 affects the stability of both TbMex67 and P34/P37 proteins, which amplifies the influence of TbMtr2. Thus, we hypothesize that within the TbMex67-TbMtr2 complex, TbMtr2 plays a more critical role than previously seen in other organisms. A functional role of TbMtr2 may be to stabilize TbMex67, thus allowing the complex to associate with ribosomal subunits. The mechanism for TbMex67 and TbMtr2 association with the ribosomal subunits needs to be investigated further.

We next examined the mature rRNAs by Northern blotting with probes directed against completely processed products and found that the steady-state levels for 18S, 5.8S, and 25/28S rRNAs were not altered upon induction for either the TbMtr2 RNAi cell line or the TbMex67 RNAi cell line (Fig. 4). Only 5S rRNA levels were decreased in the TbMex67 RNAi cells. Similar observations, where a phenotype alters ribosome biogenesis without affecting levels of mature rRNAs, have been made in the literature (26).

Although our results argue for a role of TbMtr2 and TbMex67 in export of ribosomal subunits and a concomitant loss in translation, it should be kept in mind that TbMtr2 and TbMex67 have also been implicated in the trafficking of other RNPs. A defect in nuclear export of other classes of RNAs, especially mRNAs, caused by a loss of TbMtr2 and TbMex67, could be expected to impact translation as well. However, we observe numerous aberrations in the formation of the ribosomal subunits and polysomes in parasites lacking TbMtr2 or TbMex67 (Fig. 5). Specifically, we see the presence of an additional peak between the 60S and 80S peaks, the accumulation of the 80S peak, and a loss of polysomes (Fig. 5), a phenotype previously observed in yeast expressing a mutated ScMex67 (27). These observations, taken together with the yeast data, further support a model in which TbMex67 and TbMtr2 are necessary for ribosome export and additionally help to clarify the phenotype observed in yeast.

We next examined the rRNA processing events in each of these cell lines (Fig. 6), and we found that processing intermediates in the 60S pathway accumulate upon depletion of TbMtr2 or TbMex67. The accumulated processing intermediate can be detected in the fraction of the sucrose gradient corresponding to the peak that appears upon loss of TbMtr2 and TbMex67 (Fig. 6C, asterisk). Collectively, this indicates that loss of TbMtr2 and TbMex67 prevents normal processing of rRNAs, especially those of the 60S, and that accumulation of these intermediates alters the normal composition of ribosomal subunit particles and likely contributes to an overall decrease in translational activity.

It is not yet clear whether improperly processed RNAs are competent for export. However, it is evident that successful export operates as a checkpoint for properly processed RNAs (28). In addition, the compartmentalization of processed RNA species separately from immature RNA species can serve both as a safeguard against energy expenditure on futile processes (such as the translation of improperly processed RNAs) and an opportunity for regulatory functions (such as regulated retention or export of alternatively spliced mRNAs). For mRNA, it is known that splicing factors promote export (29) and that alternative processing and export involve serine and arginine-rich proteins (SR) and NXF1 (30). Improper processing tends to lead to nuclear retention (31), and disruption of export conversely leads to the accumulation of improperly processed products, suggesting a concerted link between processing and export. Our results show that in T. brucei rRNA processing is linked to export through the activities of TbMtr2 and TbMex67. In particular, the successful processing of the large ribosomal subunit RNAs depends on wild-type steady-state abundance of TbMtr2 and TbMex67.

Taken collectively, our results suggest that TbMex67 and TbMtr2 may have both shared and functionally distinct roles in ribosome biogenesis. The loss of either TbMtr2 or TbMex67 results in morphological changes, aberrant polysome profiles, and defects in rRNA processing and ultimately cell death. Loss of TbMtr2 has a stronger effect on steady-state levels of ribosome biogenesis factors, as well as an earlier time of onset of the phenotype. This observation may be explained by the fact that TbMtr2 affects the abundance of both TbMex67 and P34/P37 proteins and that P34 and P37 are known to be involved in ribosome biogenesis and nuclear export. We hypothesize that TbMtr2 may play a regulatory role in ribosome subunit export, both through its interaction with TbMex67 and independently. We are further analyzing the interactions between TbMex67 and TbMtr2 and the 60S ribosomal subunit. Exploring these unique interactions adds to our knowledge of trypanosome-specific features that could be exploited in the identification of new drug targets.

## MATERIALS AND METHODS

### Generation of TbMex67 and TbMtr2 RNAi cell lines.

RNAi cell lines were created as previously described (13). The full-length-coding region of TbMtr2 or approximately 500 bp of the 3′ UTR of TbMex67 was inserted into p2T7-177 (Table 1). Both vectors p2T7-177-TbMex67 and p2T7-177-TbMtr2 were linearized by NotI restriction enzyme and were transfected by electroporation into T. brucei 29-13 procyclic cells (32). Cells were selected using phleomycin (2.5 μg/ml), and clonal cell lines were generated through limiting dilutions. Knockdown (RNAi) of TbMex67 or TbMtr2 protein was induced using tetracycline (Tet) (1 μg/ml). As previously described (13), these cell lines result in a significant growth defect upon Tet induction over a 3-day time course, and results are an average from three biological replicates.

**TABLE 1:** Primer sequences (5′ to 3′)

Primer name	Sequence^*a*^	
	Forward	Reverse
TbMtr2-RNAi	TAGGATCCATGTCGGAATACGCAGATTGC	CCAAGCTTTTACTCCTTTTCACTTAACCAGCG
TbMex67-RNAi	TCGGATCCTTTTTGATTCTCTGTGGTATCTGG	CCAAGCTTTGGCAACACTCTACGGTGTC
3×-Ty-TbMtr2	GTAAAGATATAAAAAGAAATATATTACATATAT ATATCAAGTGAAGGAACAGCTCTAACGACTCTC ATCACTTATTACCAgtataatgcagacctgctgc	TTATCTAACAGTTGGTAAAACTGCTGGCGAAA TCAAGCGCCTTCTTAGCCACTTCCGTGCAATCT GCGTATTCCGACATactacccgatcctgatcc
3×-Ty-TbMex67	AAAAGAAAAAAGAAGAATAGAGAAAAAAGGA AAAGAATCCAAGGCGTCAATTAATAATAAGGT GAGTTTAAAGGGTTAAAgtataatgcagacctgctgc	TTGTTCGCGCATGACCCTCTTTTAAAGTAGACA CACGGTATACGCGATGTATCCTTGTTTTTCCTGT ATGGGTTGGGCATactacccgatcctgatcc

a Lowercase sequences are pPOTV-6-specific sequences. Uppercase sequences are target-specific sequences.

### Generation of Ty-TbMex67 and Ty-TbMtr2 cell lines.

Due to the limited stock of reliable protein-specific antibodies available, 3× Ty-TbMex67 and 3× Ty-TbMtr2 gene inserts were created by PCR using a pPOTv-6 plasmid and gene-specific primers containing three copies of the Ty tag (Table 1) as previously described (32). Transfection of wild-type (427-Lister procyclic) and RNAi (29-13 procyclic) cell lines was performed as described above with 50 μl of unpurified PCR product(s). Cells were selected using puromycin (1 μg/ml), and clonal cell lines were then generated through limiting dilution.

### Ty-tagged *in vivo* immune captures.

Immune capture experiments were performed using a Ty tag-specific antibody (ThermoFisher Scientific). A whole-cell extract was created with Ty-TbMex67 and RNAi TbMtr2-TyTbMtr2 (uninduced) as previously described (33). Antibody cross-linked protein A-Dynabeads (Invitrogen) were pretreated with poly(dI-dC) for 1 h at 4°C and then were incubated with extract (1 mg/ml) overnight at 4°C. The beads were washed three times with phosphate-buffered saline (PBS) containing 0.05% Tween 20. Beads were then heated at 70°C for 10 min with SDS-PAGE sample buffer to elute off bound proteins. Western blot analysis was performed as described below, and results shown are representative of three biological replicates.

### Western blot analysis of RNAi TbMtr2-TyTbMtr2 and RNAi TbMex67-TyTbMex67 cell lines.

Cells (1 × 10^6^) were collected following RNAi induction. Sedimented cells were washed with PBS, suspended in SDS-PAGE buffer, and electrophoresed using SDS-PAGE. Western blot analysis was performed with specific antibodies as follows: P34/P37 (1:2,000; Bethyl Laboratories), TbMex67 (1:2,000; a gift from Mark Carrington, Cambridge, United Kingdom), L5 (1:2,000; Bethyl Laboratories), S5 (1:1,000; Novus Biologicals), Ty tag-specific antibody (1:2,000; ThermoFisher Scientific), and Hsp70 (loading control, 1:2,000 [34]) and His antibody (1:1,000 dilution; Invitrogen). Protein levels are normalized to the levels of Hsp70 protein for each day, and the day 3 postinduction levels are reported as average relative quantities compared to day 0 with calculated standard deviations. Densitometric quantifications were obtained using Image Lab (Bio-Rad), and results shown are representative of the three biological replicates.

### Polysome profile analysis.

Cells were sedimented, treated with cycloheximide to block translational elongation, and lysed. The lysate was cleared by centrifugation and supplemented with heparin and RNaseOUT. Extract corresponding to equal cell number was then layered onto a 10 to 40% sucrose gradient and centrifuged at 4°C for 2 h at 230,000 × *g*. Following ultracentrifugation, the gradient was fractionated and analyzed using an ISCO UV detector paired with a fraction collector. For comparison between profiles, when the *A*_260_ of the extract differed across samples, an adjustment factor was introduced in the *y* axis. At least three biological replicates were analyzed for each postinduction time point and for each cell line. Areas under the peaks were quantified using ImageJ.

### Northern blot analysis.

RNA was isolated from cells and gradient fractions using TRIzol or LS TRIzol, respectively. Five micrograms of total RNA was loaded on 1% denaturing agarose gels, electrophoresed at 100 V for 1 h, transferred to Supercharge nylon membranes (Whatman), and UV cross-linked (Stratagene UV cross-linker). RNA quality was verified by methylene blue staining. Blots were prehybridized (ThermoFisher Scientific), incubated overnight in the presence of specific radiolabeled oligonucleotide probes as previously described (20), and exposed to a phosphoscreen, which was then scanned using a phosphorimager (Typhoon). At least three biological replicates were analyzed for each postinduction time point, each cell line, and each probe.
